# Costs of Surgical Site Infections That Appear after Hospital Discharge

**DOI:** 10.3201/eid1205.051321

**Published:** 2006-05

**Authors:** Nicholas Graves, Kate Halton, Merrilyn Curtis, Shane Doidge, David Lairson, Marylou McLaws, Michael Whitby

**Affiliations:** * The Centre for Healthcare Related Infection Surveillance and Prevention, Brisbane, Queensland, Australia;; † Queensland University of Technology, Brisbane, Queensland, Australia;; ‡ University of Texas Health Science Center at Houston, Houston, Texas, USA;; § University of New South Wales, Sydney, New South Wales, Australia

**Keywords:** costs and cost analysis, surgical wound infection, community health services, economics, patient discharge, hospitalization, dispatch

## Abstract

Data were collected from surgical patients in the hospital and on 4 occasions postdischarge. The incidence of postdischarge surgical site infection was 8.46%. Strong evidence showed that these infections caused minor additional costs, which contradicts existing literature. We discuss why previous studies might have overstated costs.

Most cases of healthcare-acquired surgical site infections (SSI) appear after discharge from hospital (*1*); rates of postdischarge SSI between 2% and 14% have been reported (*2*). Little is known of the costs of postdischarge SSI, but 2studies suggest that they are large (*3**–**5*) with health services and patients incurring costs and subsequent production losses. The combination of high frequency and high cost suggests that programs that reduce the risks of postdischarge SSI should be adopted, but decision makers should assess the cost-effectiveness of additional prevention efforts. This exercise requires valid estimates of the change in costs and benefits from additional prevention programs (*6**,**7*). Understanding the costs of postdischarge SSI is therefore essential. The work completed so far is valuable but demonstrates some methodologic weaknesses. Plowman et al. (*3**,**4*) assessed only patient-reported signs and symptoms of postdischarge SSI, and Perencevich et al. (*5*) relied on routine healthcare records for diagnosis/surveillance and matched case patients with controls on only 3 confounding variables.

Our study assessed the costs of postdischarge SSI. We adopted a societal perspective and included the costs incurred by healthcare services, private costs, and production losses. The research method was chosen to address the suggested weaknesses of the studies of Plowman et al. (*3**,**4*) and Perencevich et al. (*5*).

## The Study

We recruited, in consecutive order, adults (>18 years of age) admitted to 3 Australian hospitals in 2004 for knee or hip prostheses, cardiovascular procedures, femoropopliteal bypass grafts, or abdominal procedures, including abdominal hysterectomies and lower segment caesarean sections. Four infection-control research nurses recruited participants and collected data during the hospital admission process and on 4 separate occasions after surgery by visiting the patients in their homes (data collection is illustrated in the Figure). Monetary estimates of all costs were made by multiplying frequency with a cost vector for the item of service (*9**–**12*). Production losses were estimated by comparing the presurgery level of (unwaged and waged) productive activity with the actual level of (unwaged and waged) productive activity achieved during the 4 weeks postdischarge. These losses were converted to a monetary value by using market prices for labor, approximated by average pretax earnings (*13*).

**Figure:**
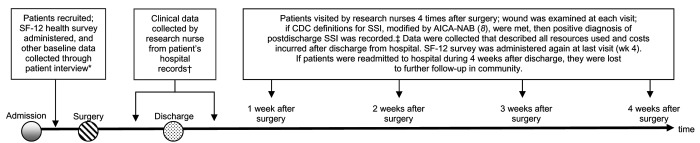
The timing and nature of data collection. *Interview questions available from author on request. †Types of data collected from patient hospital records available from author on request. ‡Variables collected from patient at each nurse visit are available from author on request. CDC, Centers for Disease Control and Prevention; SSI, surgical site infection; AICA-NAB, Australian Infection Control Association–National Advisory Board.

The question we address is whether postdischarge SSIs independently affect costs. The specific cost outcomes we seek to explain are listed in Table A1. Adjustment was made for other factors believed to influence these cost outcomes (i.e., confounding factors); these included the type of surgical procedure, duration of surgery, American Society of Anesthesiologists score, wound class, number of coexisting conditions, length of hospital stay, whether patient was funded by the public sector or private insurance, admitting hospital, sex, age, ethnicity, patient's socioeconomic status (*14*), whether the patient was in waged employment, salary level and health-related quality of life as measured by the SF-12v2 Health Survey (available from http://www.sf-36.org/tools/sf12.shtml) scores at baseline and 4 weeks postdischarge. The complete set of explanatory variables available for analyses and the summary statistics are presented in Table 1 and Table A2. Because the outcome variables were continuous and linear, ordinary least squares regression was chosen to model the independent effect of SSI on cost outcomes (Table 2). See Table A3 for a description of the statistical analyses.

**Table 1:** Demographic characteristics of included patients by surgical site infection (SSI)

Characteristic	% (no.)	
	No SSI (n = 411)	SSI (n = 38)
Age, y, mean (SD)	63.58 (14.41)	64.37 (13.72)
Socioeconomic score (1–100),* mean (SD)	35.67 (19.17)	40.37 (20.53)
Male	48.66 (199)	57.89 (22)
Recruiting hospital		
280-bed district hospital	35.04 (144)	47.37 (18)
712-bed teaching hospital	47.45 (195)	36.84 (14)
156-bed district hospital	16.79 (69)	15.79 (6)
Income		
Currently in waged employment	20.68 (85)	31.58 (12)
<$50,000/y	12.41 (51)	23.68 (9)
>$50,000/y	2.68 (11)	5.26 (2)
Refused to answer	5.35 (22)	2.63 (1)
Education		
Left school at <15 y	60.83 (250)	63.16 (24)
Left school at 16–18 y	7.54 (31)	13.16 (5)
Some form of higher education	30.41 (125)	23.68 (9)
Ethnicity		
Caucasian	96.11 (395)	97.37 (37)
Aboriginal	0.24 (1)	0.00 (0)
Asian	0.24 (1)	0.00 (0)
Other	2.43 (10)	2.63 (1)
How patient was funded		
Public	91.97 (378)	94.74 (36)
Intermediate	6.81 (28)	5.26 (2)
Private	0.24 (1)	0.00 (0)

*See Jones and McMillan (*14*) for the scoring algorithm used.

**Table 2:** Cost outcomes*

Outcome		Mean (SD)	
		No SSI, n = 411	SSI, n = 38
Healthcare services			
	No. contacts with hospital-based services in 4 wk PD	1.10 (1.68)	1.11 (1.43)
	Cost of contacts with hospital-based services in 4 wk PD ($)	40 (60)	40 (52)
	No. contacts with community-based services in 4 wk PD	1.85 (2.72)	3.13 (3.04)
	Cost of contacts with community-based services in 4 wk PD ($)	62 (103)	105 (111)
	No. tests/swabs	0.48 (1.43)	0.71 (1.27)
	Costs of tests/swabs ($)	11 (35)	16 (28)
	No. of days on antimicrobial drugs during 4 wk PD	0.96 (3.10)	6.76 (8.23)
	Costs of antimicrobial drugs	2.16 (9.08)	14.47 (19.96)
	Sum of all costs incurred by health care services, excluding costs of readmission ($)	115 (128)	176 (144)
	Sum of all costs incurred by health care services, including costs of readmission ($)	417 (3050)	2,361 (8,811)
Production losses			
	Patient production losses during 4 wk PD (min)	5,237 (5,488)	7,295 (6,349)
	Monetary valuation of patient production losses ($)	1,895 (1,986)	2,640 (2,298)
	Informal care givers production losses during 4 wk PD	1,630 (2,329)	2,863 (3,168)
	Monetary valuation of Informal care giver production losses ($)	590 (843)	1,036 (1,146)
Private costs			
	Time patient spent accessing hospital services (min)	169 (444)	184 (338)
	Time patient spent accessing community-based services (min)	129 (410)	282.76 (528.14)
	Total out-of-pocket expenditures during 4 wk PD ($)	5 (19)	4 (21)
	SF-12 Physical Component Summary (enrollment)	39.15 (11.76)	37.63 (12.24)
	SF-12 Mental Component Summary (enrollment)	50.37 (10.06)	48.87 (10.60)
	SF-12 Physical Component Summary (wk 4)	39.03 (8.84)	37.68 (8.04)
	SF-12 Mental Component Summary (wk 4)	53.92 (8.35)	52.06 (11.10)

*SSI, surgical site infection; PD, postdischarge; min, minutes of time.

The mean age of the 449 patients included in the analyses was 63.65 years (SD 14.34), and 50.56% were women. The mean length of hospital stay for the sample was 7.8 days (SD 8.68, median 6 days, interquartile range 4–8). Thirty-eight of the 449 patients included in the study had a diagnosis of SSI postdischarge, which indicates an incidence of 8.46% for the 8-month period during which patients were recruited. A higher proportion of persons with SSI (18.24%) compared to those without SSI (2.43%) were readmitted to the hospital, but the mean lengths of stay of the readmitted persons were similar, 16.57 days versus 15.72 days, respectively. Summary statistics for all variables are included in Table 1 and in Table A2, and the ICD-10 procedures for the 38 cases of SSI are described in Table A4.

No evidence was found of multicollinearity or interactions between variables. However, none of the outcome variables were normally distributed, and variance of the error term was not constant (i.e., heteroscedastic), so all models were estimated by using the Huber–White covariance matrix (*15*). Results of the ordinary least squares regressions are summarized in Table A1. Strong statistical evidence shows that postdischarge SSI independently causes the following: 1.36 extra contacts with community-based services with increased costs of $47.78; 6.46 days of additional antimicrobial drug therapy with increased costs of $14.44; and an increase in total health service costs of AU $74 (US $57) when the costs of readmission to the hospital are excluded and AU $123 (US $94) when the costs of readmission to the hospital are included. The strength of the relationship between SSI and all other cost outcomes was not significant with the 95% confidence interval crossing zero for all other models.

## Conclusions

These results support the view that most SSIs first appear after discharge from hospital, but we did not find any evidence that postdischarge SSI causes substantial economic costs even when costs are viewed from a societal perspective. These findings contradict Perencevich et al. (*5*), who found the economic cost of a case of SSI diagnosed after discharge was almost 50-fold the estimate we report here. Thus, what might explain this extreme discrepancy in attributed costs? The study designs and research methods differed. Compared to Perencevich et al. (*5*), we used more control variables (described in Table 1 and Table A2 and listed below the table in Table A1). Might this extended set of control variables reduce bias from omitted variables and so reduce the cost attributed to SSI? Another factor might be the surveillance method. Perencevich et al. (*5*) used automated record screening that relied on accurate documentation of diagnostic, testing, or treatment codes and pharmacy records. This process resulted in 89 diagnoses among 4,571 patients, an incidence rate of 1.9%. For our study, patients were recruited before surgery and infection-control research-nurses visited the patients in their homes on 4 occasions after discharge, during which time the wound was examined and the definition of the Centers for Disease Control and Prevention definition, modified by the Australian Infection Control Association Inc., was applied (*8*). This method yielded a much higher infection rate of 8.38%. One interpretation is that the surveillance method used by Perencevich et al. was not sensitive to all cases of postdischarge SSI. Instead, only those that generated certain data items in the downstream electronic records were flagged, and these may have been the most serious cases of SSI that generated the greatest costs. This theory might be supported by the higher rate of readmission among the patients with cases of SSI in the Perencevich data (34%) compared to the rate in our study (18%).

Of course, other factors may have an influence, such as the case mix and socioeconomic characteristics of the participants, the costs of the inputs to healthcare services (i.e., salaries for doctors and nurses), consumer preferences (i.e., for more or less postdischarge care), and predefined care protocols.

Also, our data only describe a 4-week period after surgery and not the 8-week period considered by Perencevich et al. (*5*). We recommend that readers interpret our results carefully but nevertheless suggest that the economic costs of SSIs that occur after hospital discharge are real but not substantial.

## Appendix

**Table A1:** Effect of surgical site infection (SSI) on cost outcomes controlling for multiple confounding factors,* the results of OLS regression estimated with robust standard errors†‡

Outcomes		Coeff. on SSI (robust st err)	95% CI lower:upper	t	p >|*t*|		R^2^
Costs incurred by healthcare services							
	No. of contacts with hospital-based services in 4 wk PD	0.44 (0.26)	–0.07:0.95	1.71	0.088		0.1996
	Cost of contacts with hospital-based services in 4 wk PD ($)	15.73 (9.46)	–2.88:34.34	1.66	0.097		0.1971
	No. of contacts with community-based services in 4 wk PD	1.36 (0.63)†	0.13:2.59	2.17	0.031		0.185
	Cost of contacts with community-based services in 4 wk PD ($)	47.78 (23.07)†	2.38:93.18	2.07	0.039		0.1809
	No. of tests/swabs	–-0.06 (0.35)	–0.75:0.62	-0.19	0.853		0.1656
	Costs of tests/swabs ($)	–3.52 (8.09)	–19.45:12.41	-0.43	0.664		0.1668
	No. of days receiving antimicrobial drugs during 4 wk PD	6.46 (1.50)†	3.51:9.41	4.31	>0.001		0.1668
	Costs of antimicrobial drugs	14.44 (3.76)†	7.04:21.84	3.84	>0.001		0.2994
	Sum of all costs incurred by healthcare services, excluding costs of readmission ($)	74.48 (29.48)†	16.48:132.49	2.53	0.012		0.2056
	Sum of all costs incurred by healthcare services, including costs of readmission ($)	123.44 (53.50)	18.17:228.71	2.31	0.022		0.1985
Production losses							
	Patient production losses during 4 wk PD (min)	699.83 (1,069.22)	–1,404.18:2,803.84	0.65	0.513		0.3935
	Monetary valuation of patient production losses ($)	253.26 (386.93)	–508.15:1,014.67	0.65	0.513		0.3935
	Informal care givers' production losses during 4 wk PD (min)	946.42 (579.55)	–193.99:2,086.83	1.63	0.103		0.2787
	Monetary valuation of informal care giver production losses ($)	342.53 (209.74)	–70.19:755.26	1.63	0.103		0.2787
Private costs incurred by patients							
	Time patient spent accessing hospital services (min)	68.57 (74.28)	–77.60:214.73	0.92		0.357	0.1056
	Time patient spent accessing community-based services (min)	180.21 (95.38)	–7.46:367.89	1.89		0.06	0.1234
	Total of out-of-pocket expenditures during 4 wk PD ($)	–3.01 (3.06)	–9.03:3.02	–0.98		0.327	0.1672
	Difference in SF-12 Mental Component Summary (enrollment vs wk 4)	–0.59 (2.32)	–5.16:3.98	–0.26		0.799	0.1653
	Difference in SF-12 Physical Component Summary (enrollment vs wk 4)	0.49 (2.69)	–4.81:5.79	0.18		0.855	0.1769

*The coefficient (coeff.) on SSI was estimated after controlling for sex, age, recruiting hospital, length of hospital stay, socioeconomic status, whether patient was in waged employment, salary level, type of surgery (International Classification of Diseases, 10th ed. system), education level, ASA, American Association of Anaesthetists, wound class, duration of surgery, number of coexisting conditions, ethnicity, whether patient was private or public, SF-12, scores at baseline and week 4 postdischarge (PD) (except for the models in which the difference in SF-12 scores is the outcome. †Statistically significant at the 5% level. ‡OLS, ordinary least squares; robust st err, robust standard error; CI, confidence interval; R^2^, relative predictive power of model; SF-12, SF-12v2 Health Survey; min, minutes of time.

**Table A2:** Clinical characteristics of included patients by surgical site infection (SSI)

Characteristics		% (no.)	
		No SSI, n = 411	SSI, n = 38
Duration of surgery (min), mean (SD)		115.89 (64.57)	130.00 (66.50)
Length of hospital stay (d), mean (SD)		7.84 (8.92)	7.39 (5.60)
ICD-10 system*			
	Blood and blood-forming organs	0.49 (2)	0.00% (0)
	Breast	2.92 (12)	5.26 (2)
	Cardiovascular	19.71 (81)	42.11 (16)
	Dental	0.24 (1)	0
	Dermatologic	0.97 (4)	0
	Digestive	13.14 (54)	13.16 (5)
	Endocrine	2.43 10)	2.63 (1)
	Gynecologic	3.65 (15)	13.16 (5)
	Male genital organs	0.73 (3)	0.00 (0)
	Musculoskeletal	46.47 (191)	18.42 (7)
	Nose, mouth, and pharynx	0.73 (3)	0
	Obstetric	3.16 (13)	5.26 (2)
	Respiratory	2.19 (9)	0
	Urinary	1.70 (7)	0
American Association of Anaesthetists			
	Not documented	2.43 (10)	0.00 (0)
	Normal healthy patient	8.76 (36)	10.53 (4)
	Mild systemic disease	47.93 (197)	42.11 (16)
	Severe systemic disease	34.06 (140)	34.21 (13)
	Severe systemic disease; patient not expected to survive	5.84 (24)	10.53 (4)
	Moribund patient	0	0
Wound			
	Clean	85.40 (351)	76.32 (29)
	Clean-contaminated	13.38 (55)	23.68 (9)
	Contaminated	0.24 (1)	0
	Dirty	0.24 (1)	0
	Unknown	0.24 (1)	0
Coexisting conditions			
	0	8.76 (36)	13.16 (5)
	1	24.33 (100)	13.16 (5)
	2	31.39 (129)	26.32 (10)
	3	17.52 (72)	13.16 (5)
	4	11.68 (48)	5.26 (2)
	>5	6.33 (26)	28.95 (11)

*ICD-10, International Classification of Diseases, 10th edition.

**Table A3:** Description of Statistical Analyses

All analyses were carried out using Stata software (Stata Statistical Software: Release 9.1, Stata Corp., College Station, TX, USA).
Evidence of multicollinearity was assessed by nested auxiliary regression, each variable was dropped from the model and the R-squared values compared to a complete model (i.e., the vif command in Stata was used to estimate variance inflation factors and the tolerances).
Nonnormality in dependent variables was assessed by visual inspection of data plots, and the Breusch-Pagan test was used as a formal test for heteroskedastic errors (i.e., the hettest command in Stata).
Terms that described interactions between socioeconomic and clinical variables were included, and the constrained model was compared with a number of unconstrained models; incremental F-tests were conducted (i.e., the test command in Stata was used).
Because the objective of the analyses was to estimate the independent effect of surgical site infections on cost outcomes, we included all available explanatory variables in a general model and did not seeks a general-to-simple reduction based on lack of statistical significance.

**Table A4:** ICD-10 procedures for each of the 38 cases of surgical site infection*

Procedure as defined by ICD-10 code	Frequency
30296-00 Total thyroidectomy	1
30321-00 Excision of retroperitoneal neuro-endocrine tumor	1
30338-00 Simple mastectomy, unilateral	1
30353-00 Extended simple mastectomy, unilateral	1
30566-00 Resection of small intestine with formation of stoma	1
30617-00 Repair of umbilical hernia	1
32024-00 High restorative anterior resection of rectum with intraperitoneal anastomosis	1
32028-00 Low restorative anterior resection of rectum with coloanal anastomosis	1
32708-01 Aorto-femoral bypass using synthetic material	1
32739-00 Femoral artery bypass using vein, above the knee	1
32754-01 Femoro-popliteal bypass using composite graft	1
35653-03 Abdominal hysterectomy with bilateral salpingo-oophorectomy	1
35717-01 Oophorectomy, bilateral	1
38477-00 Mitral valve annuloplasty with ring insertion, and 38497-02 Coronary artery bypass, using 3 saphenous vein grafts	1
38497-00 Coronary artery bypass, using 1 saphenous graft	1
38500-00 Coronary artery bypass, using 1 LIMA grafts, and 38497-01 Coronary artery bypass, using 2 saphenous vein grafts	1
38500-02 Coronary artery bypass, using 1 radial artery graft	1
49318-00 Total arthroplasty of hip, bilateral	1
16520-02 Elective lower segment caesarean section	2
33115-00 Replacement of infrarenal abdomino-aortic aneurysm with tube graft	3
33500-00 Carotid endarterectomy	3
35653-01 Total abdominal hysterectomy	3
38497-01 Coronary artery bypass, using 2 saphenous vein grafts	3
49518-00 Total arthroplasty of knee, unilateral	6
Total	38

*ICD-10, International Classification of Diseases, 10th edition; LIMA, left internal mammary artery.
